# The New York Institute of Stomatology

**Published:** 1900-01

**Authors:** 

**Affiliations:** The New York Institute of Stomatology


### THE NEW YORK INSTITUTE OF STOMATOLOGY.

A REGULAR meeting of the Institute was held on Tuesday evening, October 3, 1899, at the office of Dr. John A. Schmidt, No. 1195 Dean Street, Brooklyn, N. Y.

In the absence of the President, Dr. C. O. Kimball occupied the chair.

The minutes of the previous meeting were read and approved.

*Dr. Kimball.*—The principal subject of the evening is a paper by Dr. Wm. H. Trueman, of Philadelphia, upon "The Status of our Profession and its Educational Methods in England, as shown in Recent Publications."

(For Dr. Trueman's paper, see page 3.)

### DISCUSSION.

*Dr. S. E. Davenport.*—Dr. Trueman's paper seems a very difficult one to attempt to discuss. It is well known that Dr. Trueman is a natural student and a great reader of the literature of the profession; also that he has given much attention to dental history, and we therefore look upon him as authority upon the questions borne upon by the paper which he has read this evening.

It seems to me that he has been very skilful in bringing together so concisely the material from the scattered resources of his information. He has held up to our vision a very interesting representation of some of the sharply defined differences between the educational matters in England and in this country. It is quite evident that Dr. Trueman, while commending our English brethren for many of their ways, would place in the foreground and prefer some of the educational methods and ideas which are in use here. For one moment I almost thought I was listening to Dr. Norman W. Kingsley, when the claim that dentistry is an independent profession was brought forward so delicately. I have always felt a great interest in that claim, and I believe we all have a little leaning of preference for the independence of our profession, and yet, after all, it cannot be denied that we are only humble practitioners of a specialty of that great healing art, medicine.

What Dr. Trueman says in reference to the credit which should be given to those pioneers in our profession, for going out into the open and establishing our dental schools, emphasized the matter none too strongly, and I believe that had it not been for the efforts of those earnest men the status of dentists in England would have been on a much lower plane than it is.

We are indebted to our English brethren for many things, and we are willing to give credit to them. It seemed very natural to hear many of the names of English dentists mentioned this evening, and we are very much gratified at the prominent position taken in educational matters by the English members of this Institute.

I wish to make my acknowledgments to Dr. Trueman for the success which has attended his work upon this paper, and for coming here in person to read it.

*Dr. J. Morgan Howe.*—I am sure we are all very thankful to Dr. Trueman for his presentation of this very interesting subject, but it is so large that it is rather appalling to attempt to speak upon it without preparation. It is clear, however, from what we have learned, that the relation of the English dentist to the medical profession is quite different from that which exists here. Dr. Trueman seems to regard our position as preferable, but I am inclined to think that that is an open question, while I rather lean to the other side.

I agree with him that all honor is due to those who established dental institutions in this country, and there is no doubt, I think, that dentistry is far more advanced in this country for that bold and courageous stand that was taken; but having progressed to the state in which we find ourselves, the future rather looks to me like an increasing closer alliance with the medical profession, and that in the future, as in years gone by, we will be benefited by it.

I hardly think that the criticism that Dr. Trueman applies to the dental status on the other side of the water is necessarily connected with their relation to the medical profession. It seems to be due rather to the difficulty of legislating on the subject. Some similar difficulties have been met here, and they pertain, I should think, more to the relation of the practitioner to the public, than to his connection or relation to the medical profession; but there is much to learn by having our knowledge increased regarding the dental professional status in England. I think the paper that Dr. Trueman has been kind enough to present may be a source of great advantage to us.

*Dr. Kimball.*—It seems to me the thought that the British Association has been taking the lead in the publication of dental journals is one worthy of consideration. It may be that we have suffered in the past from an insufficient mouth-piece of the American

Dental Association, and this thought might well be in our minds, and, perhaps, bring about some change in the periodicals that are published here.

*Dr. J. G. Palmer.*—It seems strange to me that so large a society, over a thousand members, in addition to controlling a journal, should have sufficient funds invested to enable them to extend a helping hand to the unfortunate ones in our profession. We have nothing like it in this country, showing that we are too much bound up in ourselves, and lacking in brotherly kindness.

*Dr. Wm. H. Trueman.*—With your permission, Mr. President, I desire to emphasize two points referred to in my paper. First, the very large membership of the British Dental Association; only think of it! out of between four and five thousand legal practitioners, who alone are eligible, one thousand and nineteen were on the roll of this Association when it met in May, 1898. There is food for thought in this, and in the pregnant question it suggests, Why is our National Association, with a far larger field, and four or five times as many legal practitioners in its bailiwick, so poorly supported that it numbers only about one-third as many? In bringing this subject before you I had in view the lesson we might learn from the example set by our cousins on the other side. They are thoroughly organized, and loyally support their professional societies. They work together, and work in harmony; why cannot we?

The benevolent fund, to which I have referred, is seldom heard of on this side. I question, indeed, if many even know of it; yet, what a great thing it is! and so well supported. Possibly there is not the need of it here, and possibly, also, as a national affair, it may not be practicable; and yet cases have occurred when a little help from such a fund could have been deservedly bestowed, and would have been gratefully received. This fund has been from the first faithfully and well managed. I have read and followed up with much interest its yearly reports. No names are ever publicly mentioned, nor are the recipients of its bounty permitted to feel that they are charity dependents. One little incident to show its workings: A dental practitioner, who was educating his eldest son to follow in his footsteps, was cut off in the prime of life. His wife, in rearranging her affairs to meet the changed conditions and provide for her little family, found it necessary to take this boy from school and place him at an employment that would add a pittance to her resources, and it looked as though the life his father had mapped out for him was hopelessly wrecked. The managers of this benevolent fund heard of this, and at once communicated with the mother to ascertain what could be done. Surprised and gratified, she replied, that if they could allow her as much as he would earn, by straining a point she could manage to keep him at school. It was such a pittance they urged her to take more. This she absolutely refused. His education finished, through the influence of the managers of the fund, a dentist was found to accept him as a student without the usual fee, and recently, having finished his professional studies, he has, with bright prospects before him, taken his place as a member of the dental profession, and promises to be a far more useful man in the community than he ever would have been without the little help this benevolent fund so timely and kindly tendered. This is but one of many instances where this fund has stepped in, some very pathetic, and warded off the sharp sting of misfortune. Professional brotherhood is something more than a mere sentiment to men who plan and do such things. It brings and holds together the men who feel that they will not be forgotten if unfortunate, and the men who feel that it is more blessed to give than to receive.

And now the second point, the educational matters. Not living in England, and, therefore, not knowing in their entirety the ins and the outs of it, naturally lessens very greatly my ability, and perhaps my right, to pass judgment upon them; but it seems very strange to me, and I gather from reading that it is not altogether satisfactory to our English friends, that the educational matters of the dental profession in Great Britain should be so entirely in the hands of a body not only alien to the dental profession, but in many respects disposed to treat it with but little consideration or regard. During a discussion some time ago, when those portions of the curriculum that trench a little upon medicine were under discussion, one of the members of the General Medical Council arose and querulously asked, "What is this qualification you are talking about, is it a surgical qualification?" "Oh, no," the spokesman for the dental profession replied, "this is not surgery, it is dentistry, pure and simple, tooth-pulling and the like." In this same body, when these matters have been under discussion, as they have been from time to time during the last four or five years, the dental profession has been repeatedly spoken of, offensively, as a "mongrel" profession. At the session during which the new curriculum was settled, May of last year, the profession had for the first time a representative in the person of Mr. Charles S. Tomes, and it was well it had. The querulous disposition was markedly manifest.

Objection was made to the term "surgeon dentist," it was complained that a surgeon dentist was a surgeon practising dentistry, and wherever it occurred it was ordered changed to "dental practitioner." Continually the fear was expressed that by extending the dental curriculum beyond the "tooth-pulling and the like," the holder of a dental diploma would be encouraged, if his dental business was slow, to call himself a doctor, and go in for general medicine on the strength of his dental qualification. I may have been a little unjust in calling the curriculum adopted a thing of shreds and patches; but how can a systematic dental education be acquired when part of the instruction is taken with medical students in the same classes; where the teachers know nothing of and care nothing for the dental trend which alone makes their instruction valuable to a dental practitioner. Part of this course may be taken, if more convenient to the student, in a science school, part with his preceptor, part in a medical hospital, part in a dental school. They put in a great deal of time, I grant you; but how much of that time is wasted for the want of that systematic grading, blending, and interlocking; that constant care on the part of the teacher, whatever his subject may be, while not lessening by one jot the general instruction he gives, to lay especial stress upon those points which may bear more or less upon the dentist's special work, as is so faithfully done in the American Dental College. When I said that with us the dental profession was a separate and distinct calling, I did not mean that it was divorced from the great healing art, or that it had no relation to medical science; far from it. I wished to emphasize the fact that we had escaped the dilemma which has befallen our English friends. Without let or hinderance we manage our own affairs, teach our students all we think dental students should know without asking any one's permission or consent, and practise our profession as in our judgment we may best serve our clients, unembarrassed by the snarls and sneers of haughty and jealous guardians of effete prerogatives and rights. The General Medical Council of Great Britain is a mixed body. A few members are elected by a direct poll of the registered physicians; a larger number represent the great medical corporations of the kingdom; these are sufficient in numbers to control and to impress upon the council that conservatism of which they are the representatives; and a smaller number are appointed by the Crown. Mr. Tomes is a Crown appointment, and holds office for five years nominally; it is, however, practically a life appointment, and on that account esteemed more desirable and more independent. The dental profession of Great Britain is to be congratulated in having such a well-qualified representative so firmly installed.

I am impressed, and strongly so, that those critics of American dental colleges who deplore the fact that American diplomas have been barred out, and infer that this is due to anything that they have done or left undone, are sadly ignorant of the real facts in the case. When the dental profession in England, after a long and serious struggle, reached a position to assert itself, their first thought was to look after their long-neglected schools. Their best men sought the American dental colleges, and the feeling was very general that there only could a really good, practical dental education be obtained. It was not a large number that came over, compared with the number that annually joined the ranks; indeed, they were few. But the feeling was abroad that the home schools did not amount to much, and therefore a large majority of those who had not the means or the ambition to take an ocean trip rested satisfied with the instruction their preceptors were able to impart. Now, after a time, the profession in Great Britain reached a point in their upward progress when it became absolutely essential that this feeling should be changed; that the home schools should be built up and made worthy of the trust, confidence, and patronage of the profession, which in turn depended upon them for its own uplifting. Fortunately for the leaders of this movement, on glancing over the field, they discovered that of the many American dental colleges two only in their announcements called for a preliminary educational test; so by embodying this requirement in their educational plan they were able with better grace and some little show of cause to curtail this objectionable foreign competition. It was not, however, the competition itself that was the objective point. The main point was to impress upon the profession that their own home schools must be supported; that if they were not up to the mark and worthy of confidence they must make them so. The professional ranks must be recruited by educated men. They could not be so long, as the standard schools were four thousand miles away. It was a wise move, patriotic and just, and has borne excellent fruit. I was very much pleased with what I saw when shown through a dental school during my last visit to London. It was well equipped to do its part.

The barring out of foreign diplomas is not a question of education. It is a question of pounds, shillings, and pence; of dollars and cents; of francs and marks; of protection all good governments should extend to its own citizens.

*Dr. Kimball.*—We are indebted to Dr. Trueman, not only for his paper, but for the interesting discussion that he has contributed to it; and it shows the value of his preparation. His own study has brought out very carefully the points in the discussion as not one of us could have done.

We will now pass to the second paper of the evening, and I will ask Dr. Palmer to read his paper upon "Swiss Pivot Broaches: Their Peculiar Value in preparing Root-Canals."

### SWISS PIVOT BROACHES: THEIR PECULIAR VALUE IN PREPARING ROOT-CANALS.

BY JAMES G. PALMER.

These broaches are known to the majority, probably to all present, and I do not expect to present anything new. At the same time, I hope to say something that may interest you concerning what I think is their especial and peculiar value in the work of cleansing and preparing root-canals.

My preceptor, Rancil M. Streeter, was an adept in their use; and I have him to thank for what I know of how to select and use them.

They are made for jewellers' use in enlarging or reaming the fine holes in brass, for clock and watch work, and are found *hard*-tempered,—so hard that they break readily when slightly bent. (Example.)

If heated too rapidly, they will burn quickly. (Example.)

They are not round, but usually *five*-sided. These which I will show you to-night are *five*-sided, and these sides or edges are sharp.

There are several sizes, and I believe several qualities. What I will show you to-night are No. 2. The difference between Nos. 2 and 3, for instance, is slight; but between Nos. 1 and 5 considerable.

They are usually obtained in gross packages, from any dealer in jewellers' findings. There are a number of such establishments in Maiden Lane, Nassau and William Streets. Better assortments and better grades are found at such places than where all kinds of tools for mechanics are kept.

When the temper is entirely drawn and the broach rendered soft, it is very pliable, and can be tied in a knot without breaking. (Example.)

This quality enables them to follow a winding, tortuous canal with but little if any danger of breaking.

Not being round, and the angles or edges being well defined, they are good reamers, even when rendered soft, and work especially well where sulphuric acid has been introduced.

If drawing the temper entirely renders them too soft for such work in the estimation of the operator, the temper may be drawn over an alcohol lamp to suit. A spring temper can thus be obtained similar to piano-wire, or better. Much care, however, must be exercised, else the broach will be burned, as I have shown.

In a gross package one will find at least three different shapes. For the jeweller this does not make any difference, but it does to the dentist. All can be utilized; but for fine, delicate work in a root canal only one kind is desired,—that which tapers *gradually*. Perhaps half the package will consist of this kind, tapering *gradually* from the handle to the point and having sufficient thickness in the beginning of the taper to stand the strain of pushing and twisting or reaming. The other half of the package will consist of those which are light or slender all the way from the point to the handle, and of those which, while tapering well, are blunt at the point and will not go into a fine canal. Those which are too light or slender, after being made soft, will double upon themselves on slight pressure, while the thicker, blunt ones will not go more than one-third of the way into the canal.

As I have said, the temper may be drawn over an alcohol lamp to suit the operator, but to render them soft and pliable, like those which I shall show you, I have succeeded best in the following manner: Put a layer of fine asbestos fibre on a piece of sheet-iron or any convenient metal-holder; lay the broaches on this, spread out so as to avoid much contact. Then place another layer of fine asbestos over them, and, if you choose, another plate of sheet-iron over all. Place this all over a large gas-burner, or any convenient heating apparatus, and heat for an hour or more. Allow the asbestos to become *entirely* cool, and the broaches will be found uniformly soft. I find asbestos sold by H. W. Johns, 100 William Street, known as "No. 305 Fibre," to be cleaner and better for this purpose than anything I have come across.

The especial value to me of these broaches has been in cleansing and, when necessary, enlarging root-canals. I have alluded to their use for enlarging when sulphuric acid has been used. One can readily make a passage under apparently difficult circumstances, to my mind, with far less likelihood of serious results than in endeavoring to drill the canal with the engine.

In cleansing a putrid canal they are of inestimable value, because by reason of not being round, but having four and five sides, a whisp of cotton or floss silk can be wound around without slipping off, as it so frequently does from a round broach. With one of these fine, delicate broaches and just a strand of floss silk, barely perceptible, wound around it, one can go nearly to the end of the finer canals, and by frequent using of fresh silk wipe the canal as dry as one chooses. Having a number wound at one time facilitates the operation.

In the same way medicaments can readily be carried to the end of the canal, and when it is desired, by having the cotton or silk a little larger and wound tightly, the broach so wound becomes a fine piston, and will force the medicine used entirely through the foramen if desired.

A little practice in the art of winding the cotton or silk will enable one to so wind a broach that the point will be covered and protected, and will not lacerate by slipping through the silk and beyond the foramen.

In the same way they make excellent instruments to carry whatever material one may wish to fill the root with to its place. If one desires even to fill a root with gold they are valuable then, for soft foil can nicely be wound around the broach, making as long or as short a delicate cylinder as desired. This can be introduced into the canal to whatever point the operator desires, and another broach can be passed into the canal until it presses upon and against the one with the gold wound on it. Holding the second broach firmly, the first is withdrawn, and the cylinder is in place. This can be repeated until the canal is full. Do not misunderstand me. I am not advising this as a method of root-filling. It is *one* way in which it can be done.

I said the use of these broaches was not new to the dentist. Sixteen or seventeen years ago, at an afternoon and evening meeting of the Central Dental Association of New Jersey, assisted by Dr. S. C. G. Watkins, of Montclair, I gave a clinic and short description of their use.

Dr. S. G. Perry, of this city, has also written about them, and there may be others.

Notwithstanding this, I have frequently heard brother dentists express surprise when the use of a smooth broach was spoken of, and trust what I have said may be of some help. They do not take the place of the barbed broach, but are valuable adjuncts.

I have prepared a quantity, full soft, and will pass them around. These are out of a gross package recently purchased at Keller's, 64 William Street. I have assorted them so as to have the three kinds usually found in such a package separate. I have also wound some with floss silk and some with cotton for your inspection. I also show you a sample of the asbestos used. There is one lot from the original package. You are at liberty to test these,—break them, twist them, or do what you please.

*Dr. Kimball.*—We are very glad to hear what Dr. Palmer has had to say about the broaches, and shall be glad to have other points about them.

Speaking about winding broaches with cotton or silk reminds me of a little point I once received from Dr. Albert L. Buck for winding a perfectly smooth surface in making a little swab for the nose or throat. The secret is to pull the little threads of the silk or cotton apart, and wind the extreme fibres as tightly as possible. In a moment it has gotten an anchorage and readily forms a mass.

*Dr. Palmer.*—That is just the point in winding these broaches, just catching the edge of the floss at the point and beginning the winding there; but catching the floss on the fine small point of one of these Swiss broaches is a much more difficult matter than on such a broach as Dr. Buck uses.

For a long time all the barbed broaches we had were made from these pivot broaches, but there was not much care taken in selecting them nor in picking out those that were especially tapering.

*Dr. Howe.*—In regard to tempering fine broaches, I would like to call attention to the method invented or devised by Dr. Meriam, who has tempered needles and other fine instruments in glass test-tubes. I have never known of any way by which delicate instruments could be tempered so successfully.

The description of Dr. Meriam's method was published in connection with a paper on making crowns, in the INTERNATIONAL DENTAL JOURNAL for March of this year. I would advise all who are interested to read it and try his method.

These broaches which Dr. Palmer has tempered are wonderfully well treated, but Dr. Meriam's method is much simpler, and is so easily within control that one can carry it to any desired degree. Broaches or other small instruments may be put in the tube in almost any number, and the temperature raised to whatever degree desired. The change would be shown by the color of the instrument. When the proper color is reached the tube is allowed to cool off slowly.

*Dr. Davenport.*—I move that we officially thank Dr. Trueman for his paper, which is one that has taken a great deal of time and attention, and one that has sharply defined the differences between English and American methods of educating students and carrying on dental associations. Adopted.

Adjourned.

FRED. L. BOGUE, M.D., D.D.S.,
*Editor The New York Institute of Stomatology.*

